# MITIE: Simultaneous RNA-Seq-based transcript identification and quantification in multiple samples

**DOI:** 10.1093/bioinformatics/btt442

**Published:** 2013-08-25

**Authors:** Jonas Behr, André Kahles, Yi Zhong, Vipin T. Sreedharan, Philipp Drewe, Gunnar Rätsch

**Affiliations:** ^1^Computational Biology Center, Sloan-Kettering Institute, 1275 York Avenue, New York, NY 10065, USA and ^2^Friedrich Miescher Laboratory, Max Planck Society, Spemannstr. 39, 72076 Tübingen, Germany

## Abstract

**Motivation:** High-throughput sequencing of mRNA (RNA-Seq) has led to tremendous improvements in the detection of expressed genes and reconstruction of RNA transcripts. However, the extensive dynamic range of gene expression, technical limitations and biases, as well as the observed complexity of the transcriptional landscape, pose profound computational challenges for transcriptome reconstruction.

**Results:** We present the novel framework MITIE (Mixed Integer Transcript IdEntification) for simultaneous transcript reconstruction and quantification. We define a likelihood function based on the negative binomial distribution, use a regularization approach to select a few transcripts collectively explaining the observed read data and show how to find the optimal solution using Mixed Integer Programming. MITIE can (i) take advantage of known transcripts, (ii) reconstruct and quantify transcripts simultaneously in multiple samples, and (iii) resolve the location of multi-mapping reads. It is designed for genome- and assembly-based transcriptome reconstruction. We present an extensive study based on realistic simulated RNA-Seq data. When compared with state-of-the-art approaches, MITIE proves to be significantly more sensitive and overall more accurate. Moreover, MITIE yields substantial performance gains when used with multiple samples. We applied our system to 38 *Drosophila melanogaster* modENCODE RNA-Seq libraries and estimated the sensitivity of reconstructing omitted transcript annotations and the specificity with respect to annotated transcripts. Our results corroborate that a well-motivated objective paired with appropriate optimization techniques lead to significant improvements over the state-of-the-art in transcriptome reconstruction.

**Availability:** MITIE is implemented in C++ and is available from http://bioweb.me/mitie under the GPL license.

**Contact:** Jonas_Behr@web.de and raetsch@cbio.mskcc.org

**Supplementary information:** Supplementary data are available at *Bioinformatics* online.

## 1 INTRODUCTION

Most of the complexity of higher eukaryotic transcriptomes can be attributed to the encoding of multiple transcripts at a single genic locus by means of alternative splicing, transcription start and termination (e.g. Nilsen and Graveley, 2010; Rätsch *et al.*, 2007; Schweikert *et al.*, 2009). A comprehensive catalog of all transcripts encoded by a genomic locus is essential for downstream analyses that aim at a more detailed understanding of gene expression and RNA processing regulation.

RNA-Seq is a method for parallel sequencing of a large number of RNA molecules based on high-throughput sequencing technologies (ENCODE Project Consortium *et al.*, 2012; Mortazavi *et al.*, 2008; Wang *et al.*, 2009). Currently available sequencing platforms typically provide several 10–100 millions of sequence fragments (reads) with a typical length of 50–150 bases. By mapping these reads back to the genome, one can determine where gene products are encoded in the genome (e.g. Denoeud *et al.*, 2008; Guttman *et al.*, 2010; Trapnell *et al.*, 2010; Xia *et al.*, 2011) and collect evidence of RNA processing such as splicing (Bradley *et al.*, 2012; Sonnenburg *et al.*, 2007) or RNA-editing (Bahn *et al.*, 2012).

**Fig. 1.**
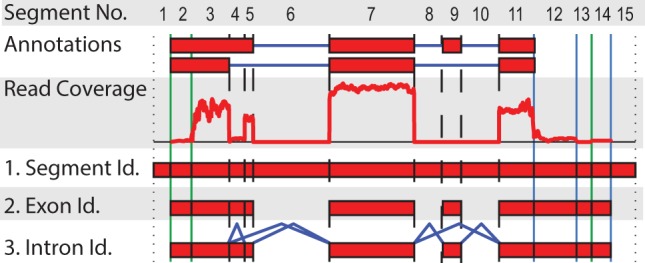
Splicing graph generation from aligned RNA-Seq reads: 1. *Segment identification:* Given a genomic region, we construct splicing graphs by generating a list of segment boundaries. Boundaries are either splice sites (SS) depicted as dashed vertical lines, potential transcription start sites (TSS) and termination sites (TTS; both depicted with solid vertical lines). Potential SS positions can originate from spliced reads (e.g. between segments 4 and 5) or annotated transcripts. Analogously, TSS and TTS sites can stem from annotated transcripts or from potential transcript end positions (e.g. between 2 and 3 as well as 13 and 14). See Supplementary Section B for more details. 2. *Exon identification:* We keep (i) segments that have >5% of their nucleotides covered, (ii) are part of annotated transcripts or (iii) if the removal of segment *s* does not leave any path between two segments connected by paired-end reads (if available). 3. *Intron identification:* We connect segments based on spliced reads and annotated introns

In many cases, the RNA-Seq reads are first aligned to a reference genome using an alignment tool that identifies possible read origins within the genome. Contiguous regions covered with read alignments (possibly with small gaps) are candidates for exonic segments. Alignment tools for RNA-Seq reads, such as *PALMapper* (De Bona *et al.*, 2008; Jean *et al.*, 2010), *TopHat* (Trapnell *et al.*, 2009), *MapSplice* (Wang *et al.*, 2010), Star (Dobin *et al.*, 2012) or *Gsnap* (Wu and Nacu, 2010) are typically able to identify new exon–exon junctions, which are candidates for introns. This information can be compiled into a segment or splicing graph, a directed acyclic graph, where the nodes correspond to exonic segments and the edges correspond to intron candidates (cf. Fig. 1 for an illustration). Assuming complete coverage, an expressed transcript corresponds to a path in the graph. Similar graphs are produced during *de novo* transcript assembly with the difference that the graph can potentially be cyclic, and the segments are not explicitly associated with a genomic location. In genome- and assembly-based transcript reconstruction, tools such as *Scripture* (Guttman *et al.*, 2010), *Cufflinks* (Trapnell *et al.*, 2010), *Trans-ABySS* (Robertson *et al.*, 2010), *Trinity* (Grabherr *et al.*, 2011) and *OASES* (Schulz *et al.*, 2012) select a subset of paths through the graph as transcript predictions. For simplicity, we will focus on genome-based transcript reconstruction when describing the approach and discuss *de novo* assembly whenever necessary.

Owing to the nature of the RNA-Seq reads, the information obtained from the alignments is of local nature only, even when considering paired-end sequencing (e.g. Smith *et al.*, 2012). The splicing graph representation implicitly assumes independence of local events. Hence, it will typically contain more paths than expressed transcripts. This is also true in the ideal case when the graph is (i) complete in the sense that it contains all vertexes and edges and (ii) accurate in the sense that it only contains expressed exonic segments as vertexes and edges that correspond to introns of expressed transcripts. For instance, the 183 807 splice variants annotated in any of the four human genome annotations (Ensembl, HAVANNA, ENCODE, Vega, see Coffey *et al.*, 2011; Flicek *et al.*, 2012; Harrow *et al.*, 2006) define splicing graphs that encode 707 386 paths, with <5% of the loci contribute >60% of the paths. Thus, we encounter a few particularly complex cases that contribute most to transcriptome complexity. Defining splicing graphs based on RNA-Seq data entails the additional difficulty that inaccurate or ambiguous read alignments can substantially increase the size of these graphs. Although this problem can be addressed by filtering the read alignments, we find that strict filtering often leads to a reduced sensitivity of transcript prediction and introduces artifacts, for instance, in the presence of unknown genomic variations.

Based on a detailed analysis of the problem and of previous work, we identified three important requirements that a transcript inference algorithm based on RNA-Seq data should meet. First, following the arguments in Xia *et al.* (2011), we note that it is important to simultaneously identify and quantify transcripts to resolve long range dependencies. In Section 4.1, we illustrate how quantitative information can perfectly deconvolve contributions from multiple transcripts, whereas ignoring quantitative information leads to inaccurate predictions. Second, enumeration of all paths defined by a splicing graph is often not tractable. For instance, for 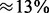 and 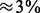 of human genes, the number of paths in splicing graphs generated from the annotation and RNA-Seq reads (see Section Supplementary Section C) is greater than 1000 and 1 000 000, respectively (see Supplementary Fig. B). This large number is the result of a combinatorial explosion of possible combinations of alternative segments and edges (This is even larger in case of *de novo* assembly where several loci are merged into cluster of connected segments if sequence is repeated). Therefore, a generally applicable approach should avoid explicit enumeration, ideally still guaranteeing optimality. Third, we show that multiple RNA-Seq samples help to solve the ill-posed problem of transcript identification (see e.g. Lacroix *et al.*, 2008; Lin *et al.*, 2012). By sharing information between samples, while still considering them separately, we can often exactly determine the correct set of expressed transcripts. We provide illustrative examples where neither merging data of multiple samples nor the independent analysis of data from each sample can solve the problem.

We describe an approach called MITIE (Mixed Integer Transcript IdEntification) that meets the aforementioned requirements. The main idea of MITIE is to report a small optimal set of transcripts that can well explain the observed RNA-Seq data in multiple samples. It does not require an explicit enumeration of all paths to find the optimal set of transcripts. This is achieved by using branch-and-bound algorithms that prune parts of the combinatorial search tree that cannot yield the optimal solution.

MITIE consists of two main parts: A data processing part generates a splicing graph from RNA-Seq alignments in Binary Sequence Alignment/Map-(BAM)-format, the annotation in Gene transfer format-(GTF)-format or both (Section 3.1). The second part solves the core optimization problem and starts with the graph decorated with quantitative information (Section 3.2). The design enables the flexible use of MITIE in existing RNA-Seq pipelines. For example, we can use the output of *Trinity*’s *inchworm* tool (Grabherr *et al.*, 2011) as input to the second part of MITIE and thereby solve the transcript reconstruction task, also solved by *Trinity*’s *butterfly* tool.

In the following section, we relate MITIE with previous work and illustrate the idea of combining multiple samples in a simulation study in Section 4.1. We then apply MITIE to a larger set of simulated RNA-Seq reads generated from the human genome annotation in Section 4.2. Finally, in Section 4.3 we analyze the performance of MITIE on a large set of RNA-Seq data for *Drosophila melanogaster* generated within the modENCODE project (Celniker *et al.*, 2009).

## 2 RELATED WORK

In this section, we discuss related work grouped by the primary goals and assumptions underlying the approaches. Approaches for genome-wide transcriptome reconstruction and quantification preceding the RNA-Seq era were mainly based on expressed sequence tags (ESTs) or microarrays. Heber *et al.* (2002) first defined and identified splicing graphs based on EST alignments, but did not devise a method to obtain transcripts from the graphs. Xing *et al.* (2004) constructed EST-based splicing graphs and called transcripts using a dynamic programming approach, preferring paths with high EST support. Wang *et al.* (2003) and Shai *et al.* (2006) identified and quantified alternative splicing events using microarray probes for exon junctions and flanking regions. Candidates were processed from annotated alternative splicing events (Wang *et al.*) or EST-alignments (Shair *et al.*), respectively. Alternative splicing events were then embedded into the reference transcript, and the resulting transcripts were finally quantified using probabilistic models for the microarray measurements. These methods constitute the algorithmic foundation of RNA-Seq based approaches, but are not directly applicable to RNA-Seq data owing to the extensive differences in abundance, error sources and biases of the underlying expression measurements.

The following RNA-Seq based methods build on quantification approaches but identify additional transcripts by enumerating all potential transcripts from a splicing graph. *iReckon* (Mezlini *et al.*, 2012) and *NSMAP* (Xia *et al.*, 2011) are capable of finding new transcripts but limit the search to transcripts having the same transcription start and termination site as known transcripts. Although this significantly reduces the search space and therefore allows simpler optimization techniques, it is a biologically implausible restriction.

*Scripture* (Guttman *et al.*, 2010) enumerates all potential transcripts from a splicing graph and reports them in the result file. Although this approach guarantees maximal sensitivity in the case of unfiltered data, it is in general not feasible, and alignments have to be filtered stringently. The approach does not aim to achieve specific results. *IsoLasso* (Li *et al.*, 2011) and *rQuant* (Bohnert *et al.*, 2009) use the *l*_1_-norm to regularize transcript abundance. This approach significantly reduces the number of reported transcripts, but the choice of the regularizer is suboptimal, given that all abundance values are positive and the sum is fixed. Thus, the regularizer does not sufficiently penalize a solution explaining the coverage with two similar transcripts compared with a solution with only one transcript (compare Mezlini *et al.*, 2012).

*CLIIQ* (Lin *et al.*, 2012) addresses this problem by applying an integer linear programing approach to limit the number of isoforms expressed in any sample combined with an *l*_1_ loss on the difference of observed and expected coverage. Athough this is conceptually similar to the MITIE optimization problem with respect to the integration of multiple samples, the formulation has significant disadvantages. The number of integer variables in the *CLIIQ* integer linear programing depends on the number of potential isoforms, which increases exponential with the number of exons. Thus, given *S* exonic segments, the theoretical runtime of the algorithm is 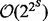, and therefore stringent filters on the read data and on the enumerated transcripts have to be applied to prevent a combinatorial explosion.

The following approaches avoid the explicit enumeration of transcripts using different techniques. *Cufflinks* reports the minimal number of transcripts such that each read alignment is explained by at least one transcript. Although this parsimony assumption reduces the computations significantly, it is violated by many known genes, and it does not deal well with inaccurate read alignments. We will discuss the benefits and drawbacks of *Cufflinks* in more detail in Sections 4.1 and 4.2. *Montebello* (Hiller and Wong, 2012) uses a probabilistic model to score sets of transcripts and implements a probabilistic search strategy to generate and modify transcript sets until a certain criterion is reached. Although this strategy allows for a wide range of functions to quantify the quality of a solution, it does not provide any guarantee of optimality. MITIE instead guides the search using the branch and bound strategy and can therefore avoid regions in the search space that cannot yield the optimal solution.

*De novo* transcript assemblers have been proven useful in cases where the reference genome is missing or of poor quality. They have the additional advantage of treating alternative transcripts and paralogous genes (resulting in multiple mappings for reads in genome alignment) naturally the same way. The optimization problem formalized by MITIE generalizes to solve transcript prediction also in the *de novo* setting, and we show in Section 4 that the MITIE strategy is superior to the dynamic programming-based strategy of *Trinity*. *OASES* follows a different heuristic, which has been shown by the authors to be more sensitive but less specific than the *Trinity* approach. *Trans-ABySS* extends the genome assembly method *ABySS* (Simpson *et al.*, 2009) to cope with the high variation in local read densities observed in RNA-Seq data. Like *Cufflinks* and *OASES*, *Trans-ABySS* does not aim to explain the read data quantitatively during the transcript prediction.

Given this context, the strategy of MITIE is comparable with quantification methods like *rQuant*, *NSMap*, *iReckon* and *IsoLasso*. The main distinctions are an improved loss function, a parsimony regularizer and the ability of MITIE to avoid an exhaustive enumeration of transcripts. The latter solves a computational problem but raises difficulties of explicitely modeling biases in the read count data with MITIE. As the improvements in quantification accuracy achieved by explicitely modeling biases have shown to be moderate [Using *rQuant* with an *l*_2_-loss function, an explicit model of the transcript length bias increased the pearson correlation coefficient by 0.4–1.7 percentage points (Bohnert, 2011, Table 3.1)], we decided not to incorporate this into our model. To our knowledge, MITIE and *Cufflinks* are the only approaches to RNA-Seq-based transcript identification that can perform predictions with and without prior annotations. We will outline the details of MITIE in the following section.

## 3 METHODS

MITIE can build a segment graph based on given alignments of RNA-Seq reads to a genome or start with segment graphs obtained by other means, in particular by *de novo* assembly. Building such graphs from RNA-Seq data has been reported several times before (e.g. Denoeud *et al.*, 2008), and we only describe it briefly in Section 3.1 and Supplementary Section B. In the following Sections 3.2–3.4, we describe (i) the main aspects of core optimization problem, (ii) how to ensure the construction of valid transcripts and (iii) give a probabilistic derivation of our loss function. We then discuss how to take advantage of paired-end reads, cope with multi-mapping reads and approximate the solution of the optimization problem in Sections 3.5 and 3.6.

### 3.1 Constructing the splicing graph

We start by defining the boundaries of a region either based on annotated genes or read coverage. If gene/transcript annotations are available, we define regions within each annotated genic locus (see Fig. 1). Otherwise, we define *islands* by identifying genomic regions that are connected by fragment alignments (cf. Supplementary Section B). Each region may contain exonic and intronic segments, and the splicing graph generation is performed independently from other regions. This process is illustrated and described in more detail in Figure 1. The main emphasis of the graph generation is completeness, whereas false information can be tolerated to some extent (see end of Section 4.3 for discussion).

### 3.2 The core optimization formulation

*Preliminaries* We define segments as sets of neighboring genomic positions corresponding to minimal entities of paths in the splicing graph 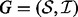 with nodes (segments) 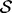 and edges (introns) 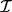. A segment *s* can be allowed to be used as the initial or terminal segment in a transcript. This information is assumed to be given as 
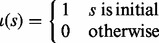
 and 
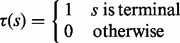
. The transcript matrix *U* is defined as a 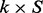 binary matrix, where 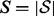 and *k* is a parameter determining the maximal number of transcripts returned by the algorithm. Paths through the splicing graph *G* can be represented as a binary vector of length *S*. Let 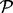 be the set of all valid paths, *R* the number of RNA-Seq samples and 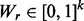 the (normalized) abundance estimates for the *k* transcripts and sample *r*. Moreover, 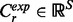 and 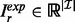 are the *expected* segment read counts and intron confirmation values (from spliced reads) for sample *r* under our model, respectively. Analogously, 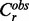 and 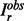 correspond to *observed* segment and intron counts for sample *r*.

*The optimization problem* Using these definitions, the core of MITIE is an optimization problem that can be formalized as:
(1)
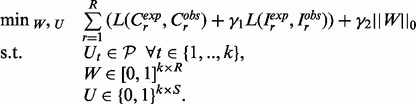

**Fig. 2.**
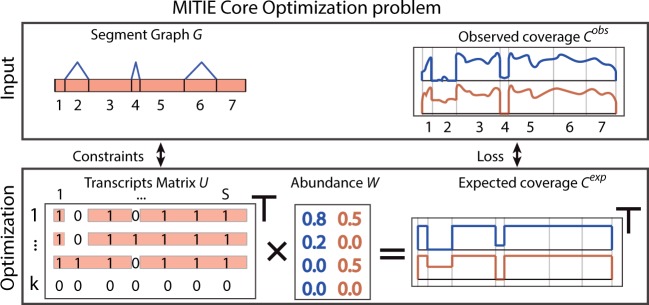
Illustration of the core optimization problem of MITIE. The transcript matrix *U* (bottom left) and abundance matrix *W* (bottom center) will be optimized such that the implied expected read coverage of the *k* valid transcripts (bottom right) matches the observed coverage (top right) well. Validity of the transcripts is ensured by appropriate constraints derived from the segment graph (top left). We illustrate the case of two samples. For each sample, we have abundance estimates *W* for each of the *k* = 4 transcripts. The identity of the transcripts, i.e., the rows of *U*, is shared among the samples. By *Occam’s razor* principle, we implement a trade-off between loss between the observed and expected coverages and the number of used transcripts, i.e. number of rows in *W* with non-zero abundances

For technical reasons, we use the normalized transcript abundance 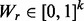. However, without loss of generality, we can define the maximal observed count as 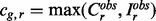, and then the expected counts can be computed from *W_r_* and *U* as 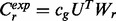. Similarly, we can compute the expected number of reads 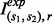 from sample *r* that span from segment *s*_1_ to segment *s*_2_ for all 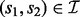 as 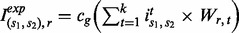, where 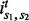 is a binary variable indicating whether intron 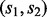 is part of transcript *t*. 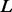 is a loss function (see Section 3.4) and 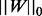 is defined as the number of non-zero rows in *W*; hence, we only count transcripts that are quantified above zero in any of the samples. 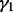 and 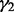 are hyper-parameters determining the trade-off between the different terms [The hyper-parameters have to be tuned by model selection to obtain the best performance. We provide useful default settings using bayesian hyper-parameter optimization strategies (Snoek *et al.*, 2012; Rasmusen and Nickisch, 2010; cf. Supplementary Section J)]. A version of this optimization problem is illustrated in Figure 2.

### 3.3 Validity of paths and known annotations

We add several constraints on *U* to make the resulting transcripts paths in the splicing graph. In particular, the constraints ensure that all feasible transcripts start at initial segments (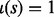), terminate at a terminal segment (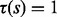) and uses only valid edges of the graph. The number of constraints is 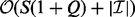, where *Q* is the average of the number of segments over-spanned by the longest intron edge starting at a specific segment *s* but is not connected to *s* (see Supplementary Section L for more details).

In cases where all transcripts are known *a priori*, we can keep the transcript matrix *U* fixed and only optimize over the abundance vector *W*. This is still a mixed integer optimization, as the sparsity term 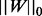 needs integer variables. If some transcripts are known and others are not, we can fix parts of the *U* matrix correspondingly. We then penalize the selection of new transcripts higher than the selection of known transcripts, thereby only predicting additional transcripts if the observed read coverage cannot be well explained by known transcripts.

### 3.4 The loss function

A commonly used loss function is the sum of squared deviations between expected and observed values (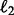-loss, see for instance, Bohnert *et al.*, 2009; Li *et al.*, 2011), i.e. 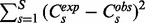. The choice of the loss function, however, reflects assumptions on the variance of the measurements (e.g. Nelder and Wedderburn, 1972). The underlying assumption of penalizing the quadratic deviation is that the measurement is Gaussian distributed with mean equal to the true abundance of the mRNA and variance constant for all expression levels. It was previously observed (e.g. Anders and Huber, 2010; Drewe *et al.*, 2012) that a negative binomial distribution with a standard deviation dependent on the mean of the observation is a better model for the distribution of read count data. We therefore make use of this distribution to define the log-likelihood-based loss function. In addition, we model background noise stemming from false alignments or incomplete RNA processing using a Poisson distribution with fixed mean λ.

We define the likelihood of observing a count *V* in dependence of the unknown expected count 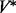 as follows

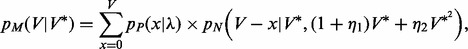

where 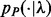 is the probability under the Poisson distribution with mean λ, and 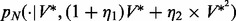 is the likelihood under the negative binomial distribution with mean 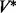 and variance 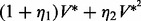. The choice for parameters 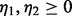 depends on the extent of biases present in the RNA-Seq library. These parameters can be estimated for a given RNA-Seq library based on single transcript genes (see Supplementary Section D for more details).

We assume deviations being independent between the segments and can thus define the negative log-likelihood 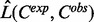 for all segments in sample *r* as follows:

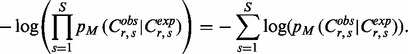



### 3.5 Exploiting paired-end reads

There are a number of ways to exploit paired-end reads. We chose a simple and efficient approach and incorporate paired end data into MITIE as follows: For each pair of segments 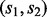, 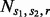 stores the number of read pairs, where one read overlaps segments *s*_1_ and the other read overlaps segment *s*_2_ in sample *r*. We then add the penalty term 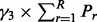 to the objective function where

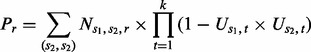

This prefers solutions in which combinations of segments supported by paired-end reads are part of a predicted transcript. A straightforward extension of this strategy also allows for integration of partial transcript information. This provides an efficient way to directly integrate information from ESTs or third-generation sequencing platforms (see e.g. Rasko *et al.*, 2011).

### 3.6 Solving the optimization problems

*Optimal solutions by mixed-integer programming* For certain classes of mixed integer optimization problems, fast solver implementations are available that guarantee to find the optimal solution. An important requirement is that the relaxed version of the problem (allowing real values for integer variables) can be solved efficiently. This allows the use of branch and bound techniques. The combinatorial tree defined by the different choices of integer variables is traversed, and on each node, the relaxed optimization problem has to be solved. As the relaxed solution is always a lower bound (in the minimization case) of the integer solution, the result of the relaxed optimization can be used to prune branches from the combinatorial tree, which cannot contain the optimal solution. In the current study, we used CPLEX (http://bioweb.me/cplex) to solve the mixed integer optimization problems.

*Piece-wise quadratic loss function* For efficient optimization, we approximate the log-likelihood term 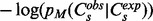 using a piece-wise quadratic proxy function 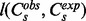 with the property that it is convex and has the same global minimum as 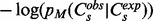 (which is reached at 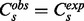). See Supplementary Section K for details on estimating 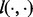. We then use this proxy function to define our loss function:

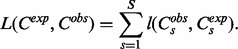



We will further refer to this loss function as the approximate negative binomial loss or 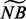
*-loss*.

*Iterative approximations* The run-time of the branch and bound algorithm depends on how widespread the near optimal solutions are in the combinatorial tree and how *branched*, i.e. complex, the tree is. The former can be addressed to a certain extend by eliminating equivalent solutions if possible (cf. Supplementary Section L). The complexity of the search tree can be reduced using constraints to exclude transcripts that are not paths in the splicing graph. However, it might become necessary to search distant parts of the tree to find the optimal solution, in particular when the number of samples is insufficient to exactly determine the solution (cf. Section 4.1). We found that we can obtain good approximations to (1), if we iteratively solve it in the following way: We first solve it for one new transcript (and available annotated ones). This speeds up computation significantly, as the number of free integer variables in *U* is *S* instead of 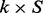. Moreover, the complexity of the combinatorial search space is significantly reduced. Once we found the first transcript, we fix the *U* variables for this transcript, keep *W* free and find a new transcript with a row of free *U* variables. This strategy significantly speeds up the optimization, and we did not notice a significant reduction in prediction accuracy.

### 3.7 Confidence quantification for transcript calls

Given a set of *k* transcripts, we are interested in the importance of each transcript for explaining the total RNA-Seq data. We make use of a likelihood-ratio test (Huelsenbeck and Crandall, 1997) to quantify the confidence in each predicted transcript *t*. We compute the test statistic:
(2)
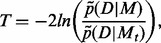

Where 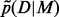 is the approximate likelihood of observing the read data *D* under our model *M* based on all *k* transcripts and 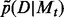 is the approximate likelihood when restricting the quantification value of transcript *t* to zero. To compute this, we solve the quantification task *k* times using all transcripts from the transcript inference step and set the quantification value of transcript *t* to zero. We compute the objective function setting all regularization parameters to zero. We assume the test statistic to be 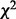
- distributed with 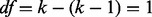 degrees of freedom and compute a *P*-value for each transcript. This strategy allows us for example to estimate the probability that a newly predicted transcript explains features of RNA-Seq data that cannot be explained by known annotated transcripts.

### 3.8 A test for differential transcript expression

Similar to the strategy in the previous section, we can also perform a test for differential transcript expression in two samples. We compute the likelihood of a model that quantifies a set of *k* transcripts independently, and the likelihood of a model that quantifies the transcripts identically in the two samples. We can then compute the log-likelihood ratio as test-statistic and apply the 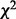-test with *df* = *k* degrees of freedom. It is straightforward to apply this test only to a subset of the transcripts. One can also extend this strategy to take replicates into account. There are a few other approaches for testing for differential transcript expression (Anders *et al.*, 2012; Drewe *et al.*, 2012; Katz *et al.*, 2010), and it goes beyond the scope of this work to provide a thorough comparison with these methods.

### 3.9 Multi-mapper optimization

The quantification of RNA-transcripts is strongly affected by reads that map to multiple locations on the genome (multi-mapped reads; cf. Section 4.2.2, for results). Predicting transcripts is even harder, and one can therefore assume that appropriate handling of multi-mapped reads can lead to significant improvements.

Our strategy is based on the multi-mapper-resolution (MMR) approach [Kahles and Rätsch personal communication: For each read with multiple possible mapping location MMR decides for the location, such that the variance in read coverage is minimized.] that decides on the read coverage distribution how to optimally choose one of several ambiguous alignments. We augmented *MMR* now referred to as *MMO* (Multi Mapper Optimization) to take the predicted transcripts and their abundance estimates into account. Given the latter, we can compute the expected read coverage throughout the genome. For each read with multiple mapping locations, we can then determine which mapping location would lead to the smallest loss over all genes in the genome [cf. (1)]. This approach has the conceptual advantage that minimizing the same loss in the core optimization step and in the multi-mapper optimization step is a consistent way of integrative RNA-Seq analysis. As *MMO* depends on transcript predictions and abundance estimates, it needs to be run multiple times in conjunction with solving the MITIE core optimization problem in an expectation-maximization-like manner. We describe more details of this approach in Supplementary Section F.

## 4 RESULTS

### 4.1 An illustrative simulation study

**Fig. 3.**
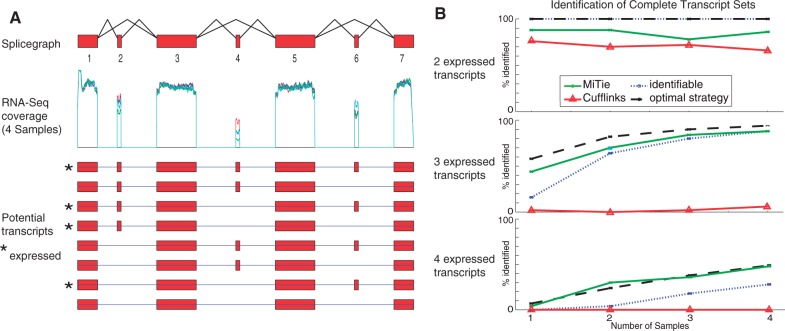
(**A**) Example with four samples of simulated reads. All four samples express the same four transcripts (marked with asterisks) with different relative abundances. The different relative abundances lead to distinct coverage patterns in the alternative regions. (**B**) We randomly selected 2 (top), 3 (middle) and 4 (bottom) transcripts and simulated four samples RNA-Seq reads each. For each sample, we uniformly redistributed the abundance between the selected transcripts. We then predicted transcripts with different methods. The prediction was counted as correct if all transcripts were exactly matched and no additional transcripts were predicted. To obtain more robust measurements, we repeated the whole procedure 50 times and report the mean number of correct predictions for each method

We start by considering a specific case of transcript inference to illustrate the limits of transcript identification from a single sample and to show how multiple samples can help identifying commonly expressed transcripts. In Figure 3A, we consider a splicing graph encoding three exons skips leading to eight possible transcripts. The task is to determine which transcripts are expressed. We consider multiple samples and assume that the same small set of transcripts is expressed in all samples but with different abundances (including the possibility of zero abundance).

This problem can be reduced to solving systems of linear equations (Lacroix *et al.*, 2008). If a system of equations is solvable, then the corresponding set of transcripts can fully explain the observed read coverage (see Supplementary Section G; for simplicity, we ignore statistical fluctuations and use exact, i.e. expected, quantities). In case of multiple samples, we identify the sets of transcripts that are consistent with all samples (intersection of the sets of sets of transcripts). If only one such set of transcripts remains, then we can be sure to have found the correct solution (‘identifiable’). If several sets remain, the best strategy is to randomly select one set out of the possible ones (‘optimal strategy’).

It turns out inference for the considered example becomes increasingly more difficult, the more transcripts are expressed (Fig. 3B). If only one transcript is expressed, all strategies always find the correct answer. If two of the eight transcripts are expressed, it is theoretically always possible to identify them correctly. Also, *Cufflinks* and MITIE often identify the correct set of transcripts (see Fig. 3B, top). Repeating the same experiment for three expressed transcripts, the observations change completely. Only in 16 and 60% of the cases, there is exactly one solution or the optimal algorithm identifies the correct one (one sample), respectively. The success rate increases significantly with the number of samples (88 and 95%). The accuracy of MITIE is close to the optimum and better than the optimal conservative algorithm. *Cufflinks* finds the correct three transcripts in only 2% of the runs, which comes close to randomly guessing three of eight (1.78%) (see Fig. 3B, middle). If four of eight transcripts are expressed, *Cufflinks* never finds the correct solution, whereas MITIE performs comparable with the optimal strategy (cf. Fig. 3B, bottom) (*Cufflinks* was run on merged samples, as the *Cufflinks*/*Cuffmerge* combination as described in Section 4.2 did perform worse).

### 4.2 Results for simulated human reads

#### 4.2.1 Read simulation

A major obstacle for the evaluation of tools for transcriptome reconstruction is the lack of a gold standard set of RNA-Seq libraries and known expressed transcripts. Simulated reads have the advantage that we can evaluate different aspects of predictions, which we would not be able to observe in reality. They are therefore an important part in evaluating many RNA-Seq-based algorithms. To obtain realistic RNA-Seq read alignments, we (i) randomly draw the transcript abundances in multiple samples, (ii) used the FluxSimulator (Griebel *et al.*, 2012) to incorporate typical biases from library preparation and sequencing, (iii) introduced errors into the generated reads and (iv) mapped the generated reads against the whole genome (see Supplementary Section A for more details). For this study, we generated simulated reads for a set of 1000 human genes with 8592 transcripts in total. The first 500 genes were used to tune hyper-parameters for all compared methods. Reported results correspond to the performance on the second set of 500 genes.

#### 4.2.2 Quantification, loss functions and multi-mapper resolution

**Fig. 4.**
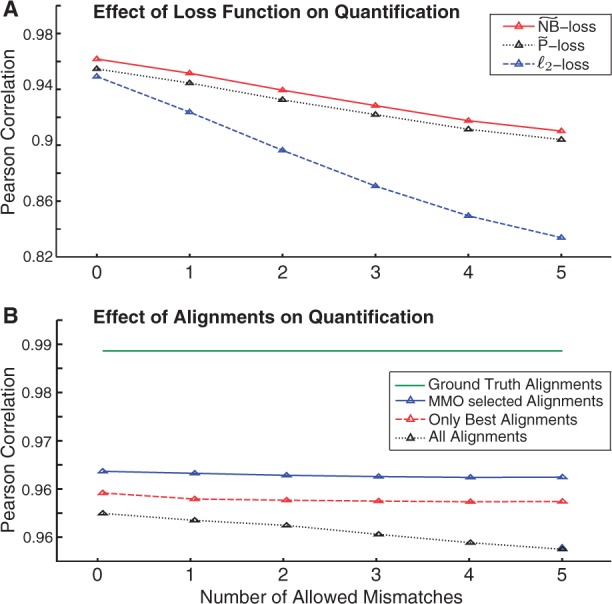
(**A**) MITIE quantification results for the three different loss functions 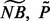 and 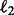-loss. We consider stringent (0 mismatches) and liberal read alignments (up to 5 mismatches), leading to fewer or more multi-mapping reads, respectively. (**B**) MITIE quantification results with 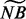-loss, when considering ground truth alignments, all multiple alignments, or after multi-mapper handling with *MMO* (see Section 3.9)

Figure 4A illustrates the effect of different loss functions on the Pearson correlation of predicted and ground truth transcript abundances. Similar to MITIE’s loss function (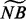-loss), we implemented a quadratic proxy function for the negative log-likelihood under the model of Poisson-distributed reads (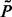-loss). The 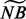-loss generally gives more accurate results than the 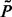-loss and the 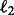-loss. Both the 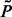-loss and 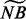-loss are significantly more robust to erroneous data (for instance, spurious alignments when allowing more mismatches) than the 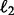-loss (For independence of hyper-parameters, we only use the exon coverage). The correlation of *Cufflinks* quantification values is significantly lower and less robust to noise (cf. Supplementary Fig. E) (We computed the correlation based on the lower confidence interval reported by *Cufflinks*, which has higher correlation to the true abundance than the estimated abundance value itself). Bohnert (2011) performed a thorough comparison of different quantification strategies including *Cufflinks* Trapnell *et al.* (2010) and *MISO* (Katz *et al.*, 2010) with and without bias correction and found that the effect of bias correction are often minor.

We also investigated the effect of multi-mapper handling on the quantification performance (Fig. 4B). For this experiment, we used the full set of MITIE features as described earlier in the text and the 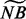-loss. We observe that using all features, the quantification is much more robust with respect to noise in the reads. Moreover, by using *MMO* (see Section 3.9), one can significantly improve the quantification. After resolving multi-mappers with *MMO*, the quantification improves even beyond less stringent filtering.

Finally, we evaluated how indicative the confidence value based on the likelihood-ratio test (Section 3.7) is for a transcript to be expressed. We find that among the 4718 non-zero quantified transcripts with *P* < 0.1, we have 86% correct transcripts with non-zero simulated expression, whereas of 326 transcripts with *P* ≥ 0.1, we find 44% correct predictions. This result shows that the confidence values accurately indicate cases with possible alternative explanations. We argue that it is more favorable to use the confidence values for filtering transcripts than the frequently used filtering based on relative or absolute abundance estimates because ambiguities might originate from the topology of the splicing graph and may therefore be independent of the expression level. This is supported by a relatively low Pearson correlation between predicted relative transcript abundance and *P*-values of only 0.18, indicating that the likelihood-ratio test adds additional information about the topology of the graph that cannot be retrieved from the predicted abundance alone.

#### 4.2.3 Accuracy of transcript prediction (MITIE and Cufflinks)

For most genes of many organisms, we know a subset of transcripts in advance. The known transcripts are likely the ones with the highest expression level as those are easiest to identify by traditional annotation strategies. To test the accuracy for this realistic scenario, we omit the information of all transcripts, except the one that has the highest simulated abundance. We ran both MITIE and *Cufflinks*, given only this one annotated transcript and the RNA-Seq reads from a larger set of transcripts to predict transcripts. We compare transcript-level sensitivity and specificity of the predictions relative to all known transcripts. We counted transcripts as being correct, if the intron structure matched the one of an annotated transcript. Single exon transcripts were counted as being correct if they overlapped with an annotated single exon transcript. Each prediction was matched to at most one annotated transcript, and each annotated transcript was associated to at most one predicted transcript (cf. Section Supplementary Section J.1).

**Fig. 5.**
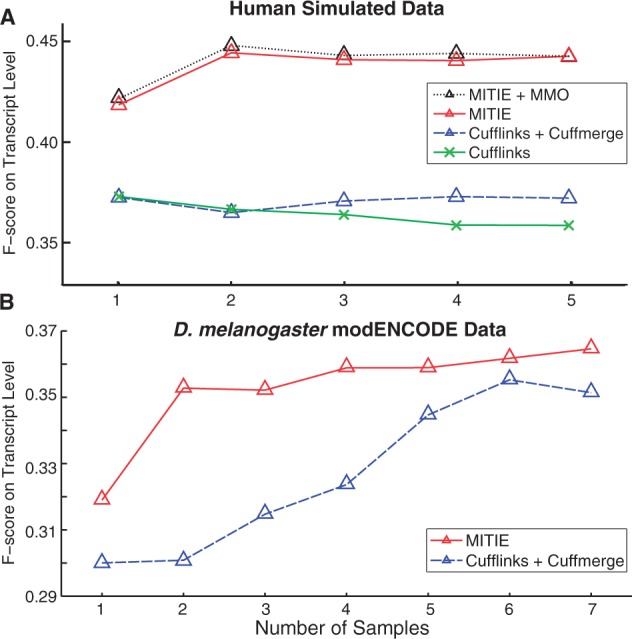
(**A**) Transcript-level F-score as a function of the number of samples for the simulated human dataset. (**B**) Transcript-level F-score as a function of the number of modENCODE samples for up to seven developmental stages of *D.melanogaster*

The results for one to five samples are shown in Figure 5A. For *Cufflinks*, we optimized the hyper-parameters (see Supplementary Section H) and used two different strategies to perform predictions. The first strategy merged all RNA-Seq alignments, and the second strategy merged individual *Cufflinks* predictions for each of the samples with *Cuffmerge*. We observe that the latter strategy outperforms the data merge strategy, but both strategies cannot benefit from additional samples. This is mostly attributed to a drastically decreasing specificity, whereas the sensitivity improves with more samples (cf. Supplementary Fig. D). MITIE with *MMO* outperforms the best *Cufflinks* prediction on average by 6.7 percentage points in F-score. MITIE/*MMO* on five samples is 2.4% more accurate than with one sample. We observe that the significant improvements *MMO* contributes in quantification accuracy to not translate to similarly high transcript recognition improvements. We attribute this to the robustness of the loss function.

#### 4.2.4 Comparison to Trinity

On the same set of simulated reads, we also compared the core optimization of MITIE to the transcript calling method *Butterfly*, which is part of the *Trinity* pipeline. We ran the entire *Trinity* pipeline and then generated MITIE predictions based on the graphs reported by the *Trinity* component *Chrysalis*.

**Fig. 6.**
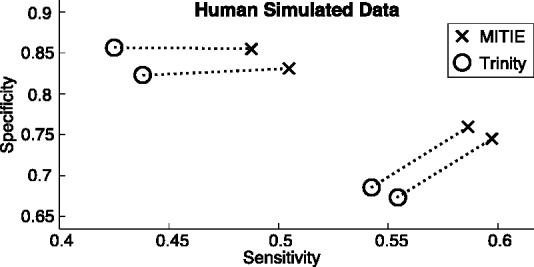
Cycles show a subset of *Trinity* model selection runs. We selected the best performing predictions for different trade-offs of sensitivity and specificity. We ran *MITIE* predictions on the De Bruijn graphs generated by trinity. Dotted lines connect the corresponding predictions

We evaluated the performance of both methods by aligning predicted mRNA sequences to the annotated mRNA sequences. A prediction was counted to be correct if (i) it was 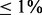 longer than the annotated transcript and (ii) the region 20 nt upstream of the first exon–exon junction to 20 nt downstream of the last exon–exon junction aligned with at most five edit operations to the reference sequence (For efficiency reasons, we ran the entire experiment for each gene separately on a FASTA file only containing the simulated reads, as they were simulated from this genic locus without mismatches.). In its current implementation, *Trinity* is not capable of integrating multiple samples. Therefore, we compared the results using only a single sample. We performed a model selection to tune hyper-parameters of *Trinity* and observed that the parameter determining the merging/splitting behavior of components (*–min_glue*) strongly influences the performance of *Trinity*. If *–min_glue = 1* predictions are more sensitive but approximately 15 percentage points less specific compared to the performance with *–min_glue = 2* (default). For both sets of predictions, we selected Pareto-optimal predictions and ran MITIE on the corresponding graphs. The MITIE core optimization problem outperforms *Butterfly* significantly in terms of sensitivity while having similar or higher specificity (cf. Fig. 6).

As *Trinity* applies stringent filtering in the *Inchworm* step, the obtained segment graph does not contain all true transcripts. From our results on genome-based assembly, we expect even higher performance gains with a more sensitive graph generation algorithm. Furthermore, we expect improvements from multiple samples, which can theoretically be used in the same way as in genome-based assembly.

### 4.3 Application to modENCODE RNA-Seq libraries

#### 4.3.1 Setup

To show that the performance improvements we have seen on simulated data translate to large-scale experimental datasets, we applied MITIE to a dataset of seven developmental stages of *D.melanogaster* (550 M alignments from 38 RNA-Seq libraries for seven developmental stages). We filtered the modENCODE *D.melanogaster* genome annotation (available from the MITIE website) for genes with at least two annotated transcripts. We then randomly removed one transcript variant (a transcript differing in splice structure to all other transcripts), which had a non-zero *Cufflinks* quantification value. We discarded genes where no such transcript could be found. From the remaining genes, we randomly selected 1000 genes for tuning the hyper-parameters and 1000 genes for testing. This setup retrospectively simulates the identification of new transcripts in already well-annotated genomes (as in Section 4.2.3).

#### 4.3.2 Results

We evaluated the sensitivity of MITIE and *Cufflinks* based on the omitted transcripts and the specificity with respect to all annotated transcripts. Figure 5B shows a comparison of the F-score as a function of the number of samples. MITIE outperforms the best (in terms of F-score) *Cufflinks* prediction in sensitivity and specificity. Similar to the simulated data, the merge of the *Cufflinks* predictions using *Cuffmerge* significantly outperforms the *Cufflinks* prediction on merged data (not shown). Although having a similar performance for large sample numbers, MITIE has a much higher F-score on up to five samples. This is mostly due to a higher sensitivity at a similar specificity.

We estimate the runtime for *Cufflinks* and MITIE for genome-wide predictions and obtain four and 19 CPU hours for one sample, respectively (see Supplementary Section E for details). Using multiple samples significantly increases the computing time as well as the accuracy. Future releases of the software will provide more efficient strategies and implementations.

As we used the alignment files provided by Celniker *et al.* (2009), we had no control over the quality or sensitivity of the alignments and multi-mapper resolution. Our results on simulated data let us expect an even higher performance for more sensitive alignments and appropriate multi-mapper handling.

## 5 CONCLUSION

The transcript prediction problem is typically under-determined. One important consequence of this observation is that deeper sequencing only helps to reduce the variance of abundance estimation and to close gaps in the splicing graph, but it does not solve the transcript identification problem as such. The proposed method reduces the set of solutions by leveraging quantitative information and multiple RNA-Seq samples combined with mild biologically plausible assumptions. Furthermore, prior information can be taken into account in a direct way within a single optimization problem, which we think will turn out particularly advantageous for integrating long reads from third-generation sequencing platforms with RNA-Seq data.

Our results highlight the importance of a well-motivated loss function to penalize the read count deviation. The application of the 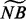-loss significantly improved our quantification and transcript recognition results, while it comes at nearly zero additional computational cost.

The underlying assumption of previously published transcript calling strategies like *Cufflinks* and *Trinity* is correctness and completeness of the graphs. Achieving both at the same time is challenging and typically not possible. This results in either wrong transcript predictions that have to be filtered out heuristically or in fragmented transcript predictions. MITIE assumes completeness of the graph, but not correctness. Completeness can often be achieved by not filtering the input alignments or not pruning the assembly graph. The decision of filtering can be deferred to the optimization problem that may choose to discard information in a context-dependent way. This is conceptually more attractive than global and uninformed filtering as a pre-processing step.

MITIE finds a solution that is compatible to the overall observed read data. As observed on simulated and real world RNA-Seq data, MITIE pushes the boundaries of what can be observed from RNA-Seq data toward more complex mixtures of transcripts by leveraging variability between samples. These improvements come with the downside of higher computational costs; however, the vast majority of cases can be optimally computed within seconds, and our implementation provides options for approximations in cases where exact computations are too expensive. Furthermore, our experiments clearly show that we can obtain the same performance as competing methods with only a fraction of the data, which in turn can save the time, money and storage capacity of deeper sequencing.

MITIE allows us to pool information from different samples in an effective way. This conceptual improvement will further future RNA-Seq studies; rather than spending efforts into deep sequencing of a few samples, future studies will have the choice to investigate a larger variety of samples at a lower depth. The combination of these samples allows us to obtain more confident transcript predictions in each sample and more insights into the biological questions at the same time.

## Supplementary Material

Supplementary Data
