# Supplementary material for: MITIE: Simultaneous RNA-Seq-based transcript identification and quantification in multiple samples
Source: Bioinformatics. 2013 Aug 25;29(20):2529–38. doi: 10.1093/bioinformatics/btt442 (PMC3789545; doi:10.1093/bioinformatics/btt442)
Supplement: Supplementary Data [file supp_29_20_2529__index.html]

MITIE: Simultaneous RNA-Seq-based Transcript Identification and Quantification in Multiple Samples — MITIE: Simultaneous RNA-Seq-based transcript identification and quantification in multiple samples — MITIE: Simultaneous RNA-Seq-based transcript identification and quantification in multiple samples — Supplementary Data 

# MITIE: Simultaneous RNA-Seq-based transcript identification and quantification in multiple samples

## Supplementary Data

files

**Files in this Data Supplement:**

- Supplementary Data - pdf file
