# Supplementary material for: MITIE: Simultaneous RNA-Seq-based transcript identification and quantification in multiple samples
Source: Bioinformatics. 2013 Aug 25;29(20):2529–38. doi: 10.1093/bioinformatics/btt442 (PMC3789545; doi:10.1093/bioinformatics/btt442)
Supplement: Supplementary Data [file supp_btt442_MAINDOC.pdf]

Supplementary Material

MITIE: Simultaneous RNA-Seq-based Transcript Identification and Quantification in Multiple Samples

Jonas Behr,[1,2] André Kahles,[1] Yi Zhong,[1] Vipin T. Sreedharan,[1] Philipp Drewe,[1] and Gunnar Rätsch [1]

[1]Computational Biology Center, Sloan-Kettering Institute, 1275 York Ave, New York, NY 10065, USA
[2]Friedrich Miescher Laboratory, Max Planck Society, Spemannstr. 39, 72076 Tübingen, Germany

SUPPL. SECTION A READ SIMULATION AND ALIGNMENT

Suppl. Section A.1 Read simulation

Based on the genes structure generated as described in Suppl. Section C, we randomly selected 1000 genes from chromosome II with more than three transcripts. The reads were simulated using the flux simulator (Griebel *et al.*, 2012). The gene expression values c_g (sum over transcript mRNA copy numbers) for gene g was given by

$$c_g = \sqrt{\frac{m}{x_g}} \times e^{-\frac{x_g}{x_1} - \frac{x_g}{x_1}^2}$$

Where $m = 10^8$, $x_1 = 2,000$ and x_g is randomly chosen without replacement from the natural numbers from 1 to x_1. Therefore, c_g values range from 60 to $9,980$ with an average of 567. Gene expression values were then distributed over the transcripts based on a stick breaking process: We randomly permuted the transcripts and the assigned a fraction δ_1 of the total mass to the first transcripts, where δ_1 is uniformly distributed between zero and one. Figure A shows the distribution of average abundance values we obtain with this strategy in comparison to the distribution of relative abundance values measured on the drosophila modENCODE data set using *Cufflinks*.

We iterate by assigning a fraction of δ_i of the remaining mass to the i-th transcript. Read length was set to 75bp, library preparation simulation parameters were chosen to be "random priming" and "chemical fragmentation".

For each of the five samples we obtain approximately 2.85 million fragments for 1000 genes, corresponding to about 57 million read for the whole human genome (assuming 20,000 expressed genes). We note that this is in the same order of magnitude as the *D. melanogaster* data set comprising 550 million reads in total. The gene structures and simulated reads are available from the MITIE website (`www.bioweb.me/mitie`).

We simulated sequencing errors by estimating an error model based on an Illumina sequencing run (HepG2 Encode cell lines, ENCODE Project Consortium *et al.*, 2012) The error model computes a mutation probability based on read quality scores, while error positions are assumed to be independent. A set of read quality strings was sampled from the same Illumina run and randomly assigned to a read. Thus, we obtain a read error distribution similar to a given Illumina run. This strategy is implemented in the Palmapper package (De Bona *et al.*, 2008; Jean *et al.*, 2010).

Suppl. Section A.2 Read Alignment

Reads were aligned against the human reference genome hg19 using PALMapper (Jean *et al.*, 2010). We performed very sensitive alignments allowing up to 10 mismatches and 2 gaps (but at most 10 edit operations in total). Splice site predictions were made with the *mGene toolbox* (Sonnenburg *et al.*, 2007; Rätsch *et al.*, 2007; Schweikert *et al.*, 2009). Not mappable reads were allowed to be trimmed to a minimum length of 40, junction remapping was allowed with a junction coverage greater than 2. All further options are summarized below:

```
-l 10 -L 20 -K 12 -C 30 -I 200000 -NI 2 -SA 100 -CT 50 -a -S -seed-hit-cancel-threshold 1000
-report-splice-sites 0.95 -filter-splice-region 5 -qpalma-use-map-max-len 2000
-qpalma-prb-offset-fix -min-spliced-segment-len 8 -report-splice-sites-top-perc 0.01
```

SUPPL. SECTION B SPLICING GRAPH GENERATION

We generate the splicing graphs in four major steps from aligned RNA-Seq reads:

1. *Genomic region identification:* For genes where at least one transcript was known we used genomic regions starting 50000 bases upstream of the transcript start to 50000 based downstream of the transcript end to account for potentially significantly longer transcript. We then trimmed the regions if we found a gap in read coverage of more than 100 bases that was also not overlapped by spliced reads. Other parts of the genome were segmented into regions based on a map adding per position coverage, number of spliced reads spanning a position and number of read-pairs spanning a position. Whenever the value of this map exceeds a user defined threshold (default 2)

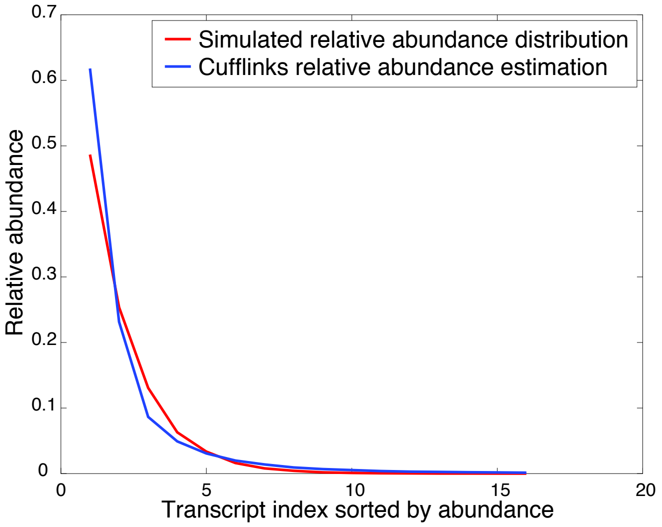

Fig. A. Relative abundance of transcripts sorted by expression. We measure the average relative abundance of transcripts in genes with at least 3 transcripts using *Cufflinks* for quantification on modENCODE data (blue). In red we show the average relative transcript abundance obtained with a uniform stick breaking process.

we call a region. We join neighboring regions with a distance of less than 50 bases and finally, we discard regions with fewer than a user defined number of reads (default 50).

2. *Segment identification:* Given a genomic region, we construct splicing graphs by generating a list of segment boundaries. Boundaries are either splice sites (SS), potential transcription start sites (TSS) and termination sites (TTS). Potential SS positions can originate from spliced reads or annotated transcripts. Analogously, TSS and TTS sites can stem from annotated transcripts or from potential transcript start and end positions inferred from RNA-Seq coverage. We identify possible start and end positions as a) drop of the read coverage to zero or b) steep drops in read coverage. The latter we find by applying a statistical test as follows. For each segment, we use a sliding window of length 60 and compare the number of read starts (ends) in the first half of the window to the corresponding number in the second half of the window in case of TSS (TTS). We apply a binomial test on the obtained counts and call a TSS/TTS site, if the p-value is smaller than 10^{-4}.
3. *Exon identification:* We keep segments that a) have more than 5% of their nucleotides covered, b) are part of annotated transcripts, or c) if the removal of segment s does not leave any path between two segments connected by paired-end reads (if available).
4. *Intron identification:* We connect segments based on spliced reads and annotated introns.

See Figure 1 for more details.

SUPPL. SECTION C NUMBER OF PATHS IN HUMAN SEGMENT GRAPH

We merged the four human genome annotations (Ensembl, HAVANNA, ENCODE, Vega, see Harrow *et al.*, 2006; Coffey *et al.*, 2011; Flicek *et al.*, 2012; ENCODE Project Consortium *et al.*, 2012) by first creating the union of all transcripts for each gene. We then merged transcripts having identical splice structure. We consolidated minor deviations in transcript ends by using the mean on the original transcript ends in the resulting transcript. We generated a splicing graph and obtained additional transcript features by integrating evidence from two RNA-Seq libraries for cell lines HepG2 (wgEncodeCshlLongRnaSeqHepg2CellLongnonpolyaAlnRep2.bam) and K562 (wgEncodeCshlLongRnaSeqK562CellPapAlnRep1.bam) from `http://hgdownload.cse.ucsc.edu/goldenPath/hg19/encodeDCC/wgEncodeCshlLongRnaSeq/` ENCODE Project Consortium *et al.* (2012). The integration was performed using the splice graph generation strategy described in Section 3.1.

SUPPL. SECTION D READ COUNT VARIABILITY WITHIN TRANSCRIPTS

To estimate the variability of read counts falling into segments we investigated human annotated single transcript genes using one RNA-Seq library from the ENCODE project (library for cell line K562 (wgEncodeCshlLongRnaSeqK562CellPapAlnRep1.bam) from `http://hgdownload.cse.ucsc.edu/goldenPath/hg19/encodeDCC/wgEncodeCshlLongRnaSeq/`) ENCODE Project Consortium *et al.* (2012). We counted read starts falling into 20 randomly selected segments for each transcript and computed mean and variance. Figure C shows a scatter plot of mean versus variance. As discussed in the main manuscript we model the relationship of mean and

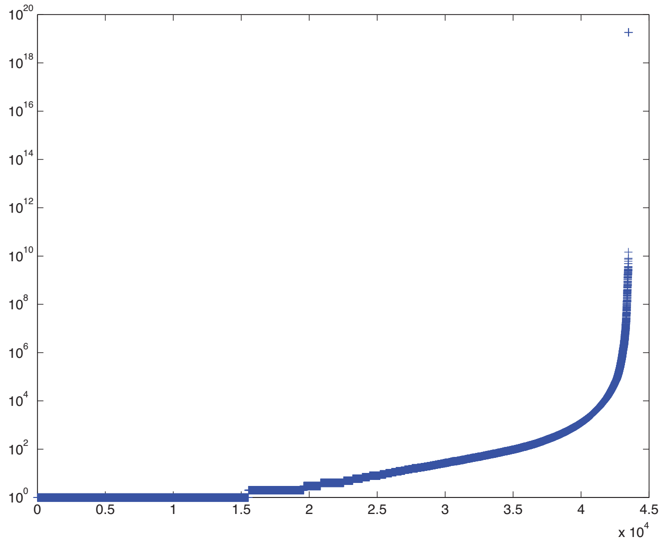

Fig. B. Number of paths in splicing graphs for 43,500 annotated human genes. We obtained the splicing graphs by adding RNA-Seq evidence from one sample to existing splicing graphs generated from annotated transcripts.

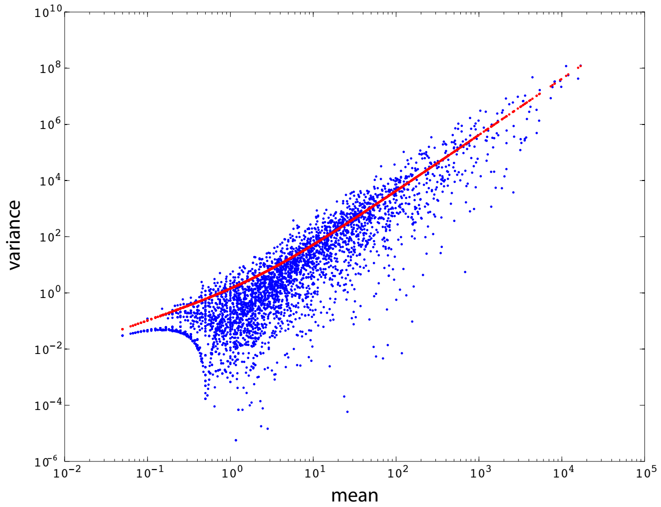

Fig. C. Relationship of mean and variance of read starts for randomly selected segments in 10,820 single transcript genes (human hg19). We counted read starts for up to 20 non-overlapping regions of length 30nt and computed mean and variance of read start counts. In red we show the weighted least squares fit.

variance with $\sigma^2 = (1+\eta_1)\mu + \eta_2\mu^2$. We estimate parameters η_1 and η_2 by computing a weighted least squares fit, where the standard least squares is modified using a gaussian distributed weighting (mean zero and standard deviation 5,000) to increase robustness. For $\eta_1 = 0$ we find that $\eta_2 = 0.42$ gives the best fit for the observed data (see Figure C red dots). We repeated the analysis for one sample obtained from the TCGA RNA-Seq data collection (`https://wiki.nci.nih.gov/display/TCGA/RNASeq+Data+Format+Specification` TCGA-DK-A3IM-01A-11R-A20F-07.d0cbd85a-e244-4f99-9adf-71f7afa5f6ec aligned with *Star aligner* (Dobin *et al.*, 2012)) and obtained an optimal value of $\eta_2 = 0.50$. We conclude that we consistently observe a significantly higher variability between segments in the same sample than previously observed for the same segment in different samples (cf. Anders and Huber (2010) and Drewe *et al.* (2012), where values for η_2 ranging from 0.1 to 0.2 were estimated). For the data sets simulated using the Flux Simulator we estimate $\eta_2 = 0.38$.

SUPPL. SECTION E RUNTIME ESTIMATION FOR THE D. MELANOGASTER MODENCODE DATA SET

We performed predictions for regions overlapping with 2000 evaluation genes. These genes have at least two expressed transcripts with different splice structure. We used *Cufflinks* to quantify transcripts in order not to bias the selection towards MITIE. Using the runtime measurements of the core optimization problem (excluding data processing steps largely depending on the file system and network speed) for those 2000 genes we estimate the total runtime by correcting for the complexity of those examples. We take the number of annotated transcripts as a proxy for the complexity and compute the α-trimmed average runtime for genes with k annotated transcripts. The α-trimmed

mean is the mean obtained after discarding the values below the α and above the $100 - \alpha$-percentile. We use $\alpha = 1\%$. This is justified since we performed computations on a subset of all genes in order to be able to compute the optimal solution in all cases. However, the very expensive cases are cases where many solutions are plausible. We observed a significant lower performance for these cases and therefore in practice it is plausible to stop computations earlier and stay with a solution, where e.g. we allow only one or zero transcripts in addition to the annotated transcripts. The loss in performance is then at most α and likely much lower. Since there are no genes with less than two transcripts in the set we use the average runtime for genes with two transcripts also for genes with one transcript. We then multiply the number of genes with k transcripts in the annotation with the average runtime observed on this subset and integrate over k to estimate the runtime for a genome wide prediction. We obtain 19.33, 560.61, 873.39, 1189.58, 1414.62, 1184.68, and 1206.07 CPU hours for one to seven samples, respectively.

For *Cufflinks* we recorded the CPU-time spend in the *assemble_bundle* method in the *cufflinks.cpp* file using the *boost::chrono* library. We recorded the time for each *bundle* and stored it with bundle start and stop coordinates in a separate output file.

SUPPL. SECTION F MITIE+MMO: JOINT-OPTIMIZATION OF TRANSCRIPT ABUNDANCE, STRUCTURE AND MULTIPLE MAPPING LOCATIONS

Given the transcript structures and abundances, we can compute the expected read coverage for segments and exon-exon junctions (C_r^{exp} and I_r^{exp}; see Section 3.2). For each alignment, the overlapping intronic and exonic segments are determined. For each segment, the mean coverage is computed with and without the respective alignment. The decision where to finally place, i.e., select, the alignment of the read is made using the $\widetilde{NB}$-loss function (see Section 3.4). It computes the loss between the observed mean coverages (with and without the currently considered alignment) and the expected coverage for the current location (see Section 3.2). For each read, all pairs of possible mapping locations are evaluated computing the loss for putting the alignment to location A and not to location B or vice versa. In each case the loss for both locations is added up to a total loss. The location with the smaller total loss is chosen. This procedure is repeated iteratively for all location pairs, for all multiple mapping reads (repeated up to five times). Furthermore, it is iterated with the core MITIE optimization to obtain updated transcripts and abundance estimations in a EM like fashion (repeated up to five times). For unstranded RNA-Seq protocols this strategy can also be utilized to optimize the strand assignment.

SUPPL. SECTION G TRANSCRIPT IDENTIFICATION WITH EXACT INFORMATION

The regions in Figure 3A summarize genomic positions sharing the same composition of overlapping transcripts. If we assume to know the number of expressed transcripts in advance (3 transcripts in this case), then we know that at least five of the unknowns are equal to zero. If we randomly select 5 unknowns and set them to zero, then this results in a system of four equations and three unknowns which is either infeasible or has exactly one solution. If it is infeasible, we know that the three remaining transcripts cannot explain the read coverage. Otherwise, we found one possible solution. We iterate this by setting all possible permutations of transcripts to zero and count the number of cases the corresponding system of equations has a solution.

SUPPL. SECTION H TRANSCRIPT PREDICTION WITH *CUFFLINKS*

Model selection for MITIE optimized the F-score on transcript level. Doing the same for *Cufflinks* results in sub-optimal predictions when samples are merged with *Cuffmerge*. Thus, the main text shows the *Cufflinks+Cuffmerge* combination, where the mean of sensitivity and specificity was optimized for *Cufflinks*. Figure D shows the sensitivity and specificity for all predictions also shown in Figure 5A and in addition the result for the F-score optimized version. We note that if we optimize the same criterion for *Cufflinks* and MITIE then MITIE predictions significantly outperform *Cufflinks* predictions in sensitivity as well as in specificity. Furthermore, we note that ranking methods based on the mean of sensitivity and specificity favors trivial and meaningless solutions. One example for such a trivial solution is to select only a single high confidence transcript prediction for the whole genome which if correct results in an mean(SN, SP) of 50%, but a F-score close to zero. For each of the eight optimized *Cufflinks* parameters we tried seven values. Thus we performed 56 predictions for each optimization criterion (112 in total). Tested values were equally distributed in log-space from default value divided by five to default value times five. All optimized parameters are shown in Table A

We combined the different *Cufflinks* predictions using *Cuffmerge* and also optimized the hyper-parameter ”–min-isoform-fraction” of the *Cuffmerge* tool. Sensitivity and specificity of the predictions are shown in (Suppl. Figure D).

For the *D. melanogaster* data set we performed *Cufflinks* predictions with default parameters. The parameter values optimized for the human artificial data are not likely to perform better in this setting, since data as well as the evaluation criterion have changed. Performing the same model selection on genome wide data was computationally prohibitive.

SUPPL. SECTION I TRANSCRIPT PREDICTION BASED ON *TRINITY* GRAPHS

We parsed the graphs and read counts for all components from files ”comp*.out” and collapsed linear portions of the graph. We then resolved cycles by removing self loops and cutting each larger cycle at the first node on the path from an initial segment which is also part of the cycle.

Table A. Optimized *Cufflinks* parameters

Parameter name	Default value	Optimized F-score	Optimized mean(SN, SP)
–min-isoform-fraction	0.1	0.1	0.29
–pre-mrna-fraction	0.15	0.26	0.26
–junc-alpha	0.001	0.001	0.001
–small-anchor-fraction	0.09	0.09	0.09
–min-frags-per-transfrag	10	10	50
–overhang-tolerance	8	8	8
–trim-3-avgcov-thresh	10	10	10
–trim-3-dropoff-frac	0.1	0.1	0.1
Cuffmerge:			
–min-isoform-fraction	0.25	0.25	0.25

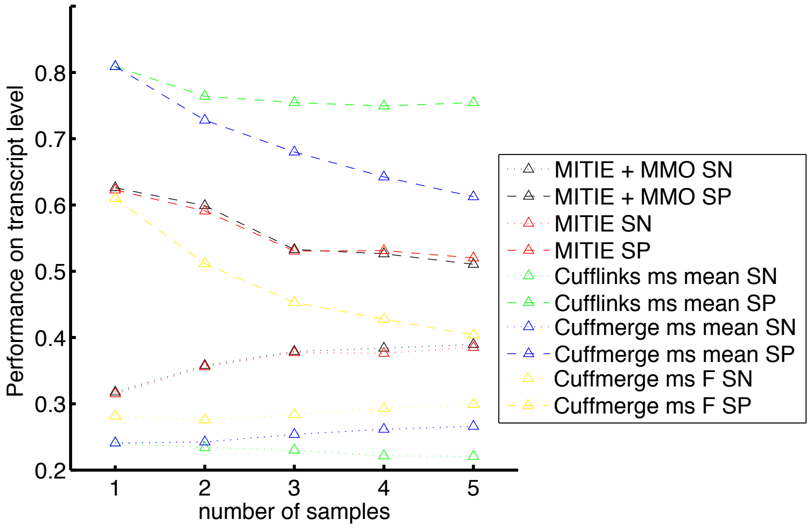

Fig. D. Sensitivity and specificity of different *Cufflinks* and MITIE predictions.

We then defined a total order of nodes ran the MITIE optimization. For simple cases with less than 9 paths we did not run the MITIE, but reported all possible paths instead.

SUPPL. SECTION J MODEL SELECTION

Following ideas from Snoek *et al.* (2012), we used Gaussian Processes (GP) to find optimal hyper-parameters for MITIE on the respective training sets. We employed the GP implementation provided by Rasmusen and Nickisch (2010). We trained a GP to predict the performance of our algorithm by randomly choosing initial parameter vectors and 20 training examples from the training set. As target values for the GP, we chose the F-score on transcript level. In each iteration, we randomly sampled parameter vectors and selected the one for evaluation. As selection criterion, we used the maximal upper confidence bound ucb:

$$ucb = \mu_y + \gamma\sigma_y$$

Where μ_y and σ_y are the mean and standard deviation of the predictive distribution of the GP. γ is a hyper-parameter of the model selection strategy and was chosen to be 2. We iterated until we had 100 data points. Finally, we selected the parameter combination with maximal lower confidence bound (lbc) to predict on the test set. The lbs can be computed as:

$$lcb = \mu_y - \gamma\sigma_y$$

The rationale behind this strategy is to explore the space and choose new parameter vectors that might lead to good performance (vectors with high predicted mean and high variance), but finally to select a vector with a high mean and low variance to achieve good performance with high confidence. We optimized the regularization parameters for (1) the number of transcripts (2) the intron coverage fit, (3) the paired-end

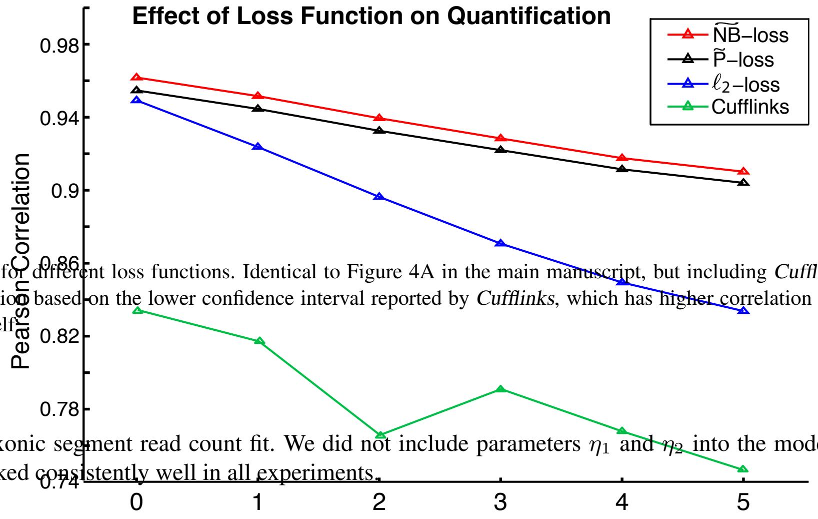

Fig. E. Quantification results for different loss functions. Identical to Figure 4A in the main manuscript, but including *Cufflinks* quantification results. We computed the Pearson correlation based on the lower confidence interval reported by *Cufflinks*, which has higher correlation to the true abundance than the estimated abundance value itself.

penalty term and (4) the exonic segment read count fit. We did not include parameters η_1 and η_2 into the model selection. We found that choices of 1.2 and 0.0 worked consistently well in all experiments.

Suppl. Section J.1 Transcript Evaluation

We evaluate against a set of annotated genes by first finding all transcripts overlapping with a given gene. We then compute a binary match score for each pair of transcripts according to the criteria described in Section 4.2.3. We then computed a maximal matching to find pairs of annotated and predicted transcripts such that the total number of correct matches is maximal. The number of matching pairs is taken as the number of true positive predictions, when computing sensitivity and specificity of the methods. Clearly, no annotated and no predicted transcript is allowed to be part of more than one such pair. All predicted transcripts not being part of a matching pair are counted as false positives and all annotated transcripts not being part of a matching pair are counted as false negatives.

SUPPL. SECTION K APPROXIMATION OF THE LOSS FUNCTION

Our optimization formulation is restricted to polynomials of degree two. Thus, we cannot directly employ the loss function L. Instead, we utilize a approximation of by fitting the this function with two polynomial of degree two. We fit L for expected values smaller than the observed value V separately from expected values larger than V. We minimize the squared error between the quadratic proxy function and the negative log likelihood function on a grid ranging from -10 to $+10$ standard deviations of the negative binomial with a step size of $V/500 + 0.002$. We obtain four coefficients ll_V(left linear), lq_V(left quadratic), rl_V(right linear) and rq_V(right quadratic). The resulting loss function can be written as:

$$l(V^*, V) = \begin{cases} lq_V \times (V^{exp} - V)^2 & V^{exp} < V \\ \quad +ll_V \times |V^{exp} - V|, & \\ & \\ rq_V \times (V^{exp} - V)^2 & V^{exp} \geq V \\ \quad +rl_V \times |V^{exp} - V|, & \end{cases}$$

We precompute the coefficients of l for values of V ranging from 1 to 30000. Between the positions of this grid we linearly interpolate the coefficients.

SUPPL. SECTION L CONSTRAINTS

Computing the expected segment count C^{exp} equivalent to $C^{exp}_{s,t,r} = U_{s,t} \times W_t$ [8]:

$$\begin{aligned} C^{exp}_{s,t,r} &\leq U_{s,t} & (3)\\ C^{exp}_{s,t,r} &\leq -U_{s,t} - W_{t,r} + 1 & (4)\\ C^{exp}_{s,t,r} &\geq U_{s,t} + W_{t,r} - 1 & (5) \end{aligned}$$

We require the transcript abundance $W_{t,r}$ to be zero for each sample r if transcript t does not have any segments:

$$W_{t,r} \leq \sum_s U_{s,t} \quad \forall 1 \leq r \leq R \tag{6}$$

We sort the transcript in order to reduce the search space by eliminating equivalent solutions corresponding to permutations of transcripts.

$$W_{t,1} \geq W_{t+1,1}, \quad \forall 1 \leq t < k \tag{7}$$

The transcript indicator variables $I_t = \begin{cases} 1 & \sum_{r=0}^{R} W_{t,r} > 0 \\ 0 & \text{else} \end{cases}$ can be computed as:

$$\sum_{r=1}^{R} W_{t,r} \leq R \times I_t \tag{8}$$

$\gamma_2 \times \sum_{t=1}^{k} I_t$ is part of the objective function. We enforce all predicted introns to correspond to connections in the graph $G = (\mathcal{S}, \mathcal{I})$. This can be done by firstly enforcing that, if segment s is used in transcript t (i.e., $U_{s,t} > 0$) any of the segments preceding s in the graph is used as well:

$$U_{s,t} \leq \sum_{x \in \{x|(s,x) \in \mathcal{I}\}} U_{xt} \tag{9}$$

$$U_{s,t} \leq \sum_{x \in \{x|(x,s) \in \mathcal{I}\}} U_{xt} \tag{10}$$

Secondly, we need to make sure, that no intron $(s, s2)$ is used which is not in G:

$$U_{s,t} + U_{s_2,t} <= 1 + \sum_{i=s_1+1}^{s_2-1} U_{i,t} \tag{11}$$

Since constraints (10) force any of the segments directly preceding s to be used, there is no need to exclude intron (s, s_3) for any $s_3 > max(\{i|(s,i) \in \mathcal{I}\})$. If s is a potential TSS or TTS site, do not force to use any connected upstream or downstream segment, respectively. Therefore, we have to exclude all invalid connections accordingly.

Compute expected intron count $C^{I,exp}_{s_1,s_2,t}$,. Determine if intron is used and if so let $C^{I,exp}_{s_1,s_2,t}$ be equal to W_t:

$$C^{I,exp}_{s_1,s_2,t} = W_t \times U_{s_1,t} \times U_{s_2,t} \times \prod_{i=s1+1}^{s_2-1} (1 - U_{i,t}) \tag{12}$$

This relationship can be expressed in terms of linear constraints as follows:

$$\begin{aligned} C^{I,exp}_{s_1,s_2,t} &\leq U_{s_1,t} & (13)\\ C^{I,exp}_{s_1,s_2,t} &\leq U_{s_2,t} & (14)\\ C^{I,exp}_{s_1,s_2,t} &\leq 1 - U_{i,t} \quad \forall s_1 < i < s_2 & (15)\\ C^{I,exp}_{s_1,s_2,t} &\leq W_t - U_{s_1,t} - U_{s_2,t} + 2 + \sum_{i=s_1+1}^{s_2-1} U_{i,t} & (16)\\ C^{I,exp}_{s_1,s_2,t} &\geq W_t + U_{s_1,t} + U_{s_2,t} - 2 - \sum_{i=s_1+1}^{s_2-1} U_{i,t} & (17)\\ & & (18) \end{aligned}$$

[8] For simplicity we discard the constant scaling factor c_g in the following formulation. Replace C^{exp} by $\frac{C^{exp}}{c_g}$

Paired-end information: Compute binary variable $P_{s_1,s_2,t}$ indicating that segment pair $(s1, s_2)$ is part of a expressed transcript:

$$P_{s_1,s_2,t} = U_{s_1,t} * U_{s_2,t} * I_t \tag{19}$$

In terms of linear constraints this can be rewritten as:

$$P_{s_1,s_2,t} \leq 1/3(U_{s_1,t} + U_{s_2,t} + I_t) \tag{20}$$

$$P_{s_1,s_2,t} \geq U_{s_1,t} + U_{s_2,t} + I_t - 2 \tag{21}$$

Compute binary variable $P^{any}_{s1,s2}$ indicating weather segments s_1 and s_2 confirmed by paired-end reads do not occur together in any transcript:

$$P^{any}_{s_1,s_2} \geq -\sum_{t=1}^{k} P_{s_1,s_2,t} + 1 \tag{22}$$

(23)

The term $N_{s_1,s_2} \times P^{any}_{s_1,s_2}$ is part of the objective function, where N_{s_1,s_2} is the number of paired-end fragments supporting the connection between segments s_1 and s_2.
